# Leukocyte Telomere Length Variants Are Independently Associated with Survival of Patients with Colorectal Cancer

**DOI:** 10.3390/cancers18030490

**Published:** 2026-02-02

**Authors:** Gobinda Sarkar, Jun Chen, Shubham Sood, Karen Fischer, Kim Kossick, Daniel Schupack, Rondell Graham, Brooke Druliner, Zahra Heydari, Lauren Helgeson, Estela Cruz Garcia, Richard Cawthon, Lisa Boardman

**Affiliations:** 1 Department of Gastroenterology and Hepatology, Mayo Clinic, Rochester, MN 55905, USA; sood.shubham@hcahealthcare.com (S.S.); schupack.daniel@mayo.edu (D.S.); druliner.brooke@mayo.edu (B.D.); heydari.zahra@mayo.edu (Z.H.); helgeson.lauren@mayo.edu (L.H.); estela.cruz1@upr.edu (E.C.G.); 2 Department of Computational Biology, Mayo Clinic, Rochester, MN 55905, USA; chen.jun2@mayo.edu; 3 Clinical Trials and Biostatistics, Mayo Clinic, Rochester, MN 55905, USA; 4 Department of Laboratory Medicine and Pathology, Mayo Clinic, Rochester, MN 55905, USA; graham.rondell@mayo.edu; 5 Medical Sciences Campus, University of Puerto Rico, San Juan, PR 00921, USA; 6 Department of Human Genetics, University of Utah, Salt Lake City, UT 84108, USA; rcawthon@genetics.utah.edu

**Keywords:** telomere length, colorectal cancer, survival, biomarker, allele sum, *TERC*, *OBFC1*

## Abstract

**Background and aims**: Aberrations in telomere length can have important implications in cancer. Using a cohort of 1007 patients, we investigated whether leukocyte telomere length (LTL) in patients with colorectal cancer (CRC) is associated with survival. We also investigated whether some telomere maintenance genes are associated with survival in these patients. **Methods:** The Biobank for Gastrointestinal Health Research (BGHR), an ongoing project involving collection of biospecimens at the Mayo Clinic, was utilized to obtain data from patients diagnosed with stage II or III CRC. Blood samples were collected prior to chemotherapy/radiation and DNA was extracted for measuring median LTL. The main outcome measures were overall survival (OS) and disease-free survival (DFS) by disease stage. **Results:** A significant inverse relationship was observed with patient age and LTL (spearman correlation coefficient (r) = −0.48, 95%; *p* = 1.13 × 10^−58^). Females had significantly longer LTL than males (*p* = 3.97 × 10^−5^). The rs1317082 SNP in the *TERC* gene was significantly associated with both OS and DFS in combined stage II and stage III patients (*p* = 0.017 and *p* = 0.023, respectively). A statistically significant association of the *OBFC1* SNP (rs9419958) was observed for OS for the combined stage II and stage III patients (*p* = 0.016). Importantly, LTL was significantly associated with both OS and DFS (*p* = 0.008 and 0.044 respectively) in combined stage II and stage III patients. **Conclusions:** Our results show that LTL is predictive of OS and DFS for stage II and III CRC patients, particularly over a longer follow-up, extending beyond five years after a diagnosis of CRC, and certain SNPs in genes involved in telomere maintenance are significantly associated with patient outcomes, independent of telomere length.

## Simple Summary

Telomere length is a well-known determinant of cell health and metabolic status. In general, normal cells have longer telomeres than cancer cells. Importantly, it has been shown that peripheral blood leukocyte telomere length (LTL) can be representative of the overall telomere length of an individual and has shown association with multiple age-related conditions, including cancers. With this rationale, we set out to investigate whether there is any relationship between telomere length and survival of patients with CRC. Our results show that there is a significant relationship between survival and LTL. We also show that alleles of the telomere length maintenance genes *TERC* and *OBFC1* are associated with survival from CRC.

## 1. Introduction

Despite advances in prediction models and management strategies for patients with colorectal cancer (CRC), it remains the second leading cause of cancer death in the United States [1] and worldwide [2]. Accurate prediction of risk for cancer recurrence and survival is essential for physicians to devise tailored management strategies and determine risk to benefit ratio of treatments and to enable patients to make informed decisions about their treatment options. The American Joint Committee on Cancer (AJCC) utilizes tumor extent (T), nodal involvement (N), and metastasis to distant locations (M) to determine CRC prognosis and guide management strategies (TNM staging) [3,4,5]. Approximately half of CRC patients are diagnosed at stage III, and tumor stage alone predicts overall survival (OS) and disease-free survival (DFS), but only in a little over half of the cases [6]. Currently, there is a lack of a dependable (blood-based) molecular marker for prediction of outcomes in patients undergoing treatment for CRC. Although plasma CEA levels can help to monitor response to treatment and identify tumor recurrence after surgical resection, they have low sensitivity and specificity (35% and 87%, respectively) [7]. Methylation and neutrophil-to-lymphocyte ratio have also been proposed as markers for CRC prognostication that have not been integrated into clinical practice [8,9]. The use of circulating tumor DNA (CtDNA) is another technology that is emerging [10,11,12,13,14,15]. Identification of new biomarkers is, therefore, necessary for prognostication of a greater number of patients.

Human telomeres are tandem repeats of TTAGGG nucleotides positioned at the chromosomal ends that function to prevent the degradation and destabilization of chromosomes [16]. Telomeres undergo attrition by losing approximately 50–200 base pairs after each cell division, thus serving as a marker of the biological age of an individual [17]. Further, several lifestyle factors (smoking, obesity, stress) [18,19,20] and host characteristic 74 (male sex) play a role in shortening telomere length [21]. Numerous studies have shown association of telomere length with modifiable factors like nutrition [22], vitamin D levels [23], body mass index (BMI) [24], smoking [18], and stress [20]. Interestingly, peripheral blood leukocyte telomere length (LTL) can serve as an overall indicator of the telomere length of an individual and has shown association with multiple age-related conditions, including cancers [25,26]. Studies in patients with lung, kidney, and bladder cancer have found that LTL may predict survival outcomes [27,28,29], while similar studies in CRC patients have produced equivocal results [30,31,32].

Since telomere length (TL) is a well-known indicator of both normal and cancer cell health [33], we hypothesize that LTL status will be informative as a disease prognostication marker for OS and/or DFS for CRC patients at various stages of the disease.

In addition to lifestyle and demographic factors, the genotype of an individual also determines telomere length. Certain single-nucleotide polymorphisms (SNPs) in the *TERT* (telomerase reverse transcriptase), *TERC* (telomerase RNA component) and *OBFC1* (oligonucleotide/oligosaccharide binding fold containing 1) genes have been shown to be highly associated with shorter telomere length in various studies [34,35], independently of factors like age, gender, smoking, alcohol, and BMI [36]. It has also been shown that shorter telomeres in the presence of these SNPs are associated with decreased cancer mortality [36]. The potential regulatory influence of these SNPs on leukocyte telomere length and outcomes for patients with CRC is currently unknown.

We investigated whether there is any relationship between LTL and the above SNPs and survival outcomes of stage II and stage III CRC patients. Our results indicate that LTL is indeed associated with CRC patient survival, even when our TL measurements are adjusted for age and sex, factors that are known to affect LTL.

## 2. Methods

This study was performed in accordance with the Declaration of Helsinki and following Mayo Institutional Review (IRB) approval for the project “Individualizing colorectal cancer patient care using the host and tumor telomere phenotype” (March 2016–present, IRB 15-009260) and utilizing biospecimens from patients collected through the Biobank for Gastrointestinal Health Research (BGHR), an ongoing project involving collection of biospecimens from patients undergoing normal colonoscopy examinations, removal of colorectal polyps or cancer at Mayo Clinic in Rochester (April 2000–present, IRB 622-00). One thousand and seven patients diagnosed with stage II or III CRC between 2000 and 2017 were included in this study. Clinical and demographic details were obtained through a self-administered questionnaire and abstracted from medical records. Only participants above 18 years of age who had stage II or III CRC and who had a blood sample that was chemo/radiotherapy-naïve and collected prior to surgery were included.

### 2.1. Blood Sample Collection

Blood specimens were collected in an EDTA-coated tube. The samples were maintained at 80 degrees Celsius. Separation of the sample into red blood cells, plasma, and buffy coat layer was performed at the Biospecimens Accessioning and Processing (BAP) facility at Mayo.

### 2.2. DNA Extraction and Telomere Length Measurement

DNA was extracted from the buffy coat using the Promega Maxwell RSC technology (Promega, Madison, WI, USA) and quantified using a Qubit Fluorometer (Invitrogen, ThermoFisher Scientific, Waltham, MA, USA). Telomere length was assessed from these DNA samples in triplicate using monochrome multiplex PCR reaction (mm qPCR) for measurement of telomere length [37] by the technique developed by Cawthon. The method is based on the principle of determining the copy number (Ct value) of the telomeric repeat and comparing that to the Ct value of a single-copy gene. The same amount of DNA sample was used for each PCR reaction. The technique uses two primers designed to hybridize the telomeric hexamer repeats and determine the sample’s telomere repeat copy number (T), and two other primers designed to hybridize to the single-copy gene B2 globin to produce the copy number value of the reference DNA sample (S) to subsequently produce a T/S ratio. Each T and S sample was run in triplicate. The median T/S value corresponding to a sample was representative of the telomere length of that sample. On each PCR plate, triplicate T and S copy number was assessed from a negative water control and a positive control of leukocyte DNA from a healthy participant to IRB 622-00, with an interassay coefficient of variation < 1%.

### 2.3. Genotyping Methods

DNA samples were genotyped for three SNPs in the *TERT*, *TERC*, and *OBFC1* genes. These SNPs have previously been used in the Danish Central Person Registry, where the SNPs were found to be associated with the largest effect size for shorter telomere length. Shorter telomeres in the presence of these genetic variations were associated with reduced mortality from cancer in a study of 64,000 subjects by Rode et al. [36]. These SNPs include rs7726159, a SNP in the *TERT* gene which encodes the telomerase reverse transcriptase; rs1317082, a SNP near *TERC*, which encodes the telomerase RNA template; and rs2487999, a SNP near *OBFC1* gene that is involved in the CST complex, which is a regulator of telomerase. Genotyping with the TaqMan assay (Applied Biosystems/ThermoFisher Scientific, Waltham, MA, USA) was completed according to the manufacturer’s instructions at the Institutional Core Facility at Mayo Clinic (Stabile Genomic Analysis core QS-7 Flex; Mayo Clinic, Rochester, MN, USA). Following PCR amplification, end reactions were read on ABI Prism 7900 HT using Sequence Detection Software v.2.4 1 (Applied Biosystems/ThermoFisher Scientific, Waltham, MA, USA) and Illumina Custom GoldenGate (Illumina, San Diego, CA, USA) genotyping completed. Arrays were read with Illumina Bead Array Reader and Data Analyzed with Bead Studio. Genotyping call rates and concordance with blinded duplicates were 100% each. Three negative and one positive CEPH control was run on each 384-well plate. No samples failed genotyping. Allele sums were calculated as had been performed by Rode et al. Minor allele frequencies for rs7726159, rs1317082 and rs2487999 are 34.2%, 22.5% and 10.4%, respectively. Hardy–Weinberg equilibrium among samples was not violated for any of these 3 SNPs. However, allele sum (SNP sum) of 0, 1 and 2 were categorized as a single variable due to a smaller number of patients with these values. Similarly, allele sums of 5 and 6 were also categorized as one variable.

### 2.4. Outcome Variables

Overall survival (OS) represented the time from patient diagnosis to date of patient death or date of last follow-up, whichever came first. Disease free-survival (DFS) event was defined as the first CRC recurrence or death from CRC if no recurrence was recorded, and others were treated as lost to follow-up. Recurrence was defined as diagnosis of CRC any time after treatment.

### 2.5. Statistical Analyses

Fisher’s exact test or the χ^2^ test was used for categorical variables, and two-sample *t*-test or Wilcoxon rank sum test was used for continuous variables when comparing patient characteristics. The relationship of telomere length and age was tested using a linear regression model, and differences by sex, cancer stage, and allelic groups were tested by two-sample *t*-test or ANOVA. Multiple-variable Cox proportional hazards models were used to test the independent effect of variables (genotype, telomere length, age, sex, stage) with patient survival outcomes (OS, DFS). Results were quantified as hazard ratio with 95% confidence intervals. Kaplan–Meier curves were used to compare the survival differences between patient groups and log-rank test was used for assessing significance. A *p*-value of less than 0.05 was considered statistically significant. The T/S ratio was transformed as log to obtain a normalized distribution for analysis. All statistical analyses were performed under R version 4.3.2.

## 3. Results

Baseline characteristics of the study population are shown in Table 1. Of the total cohort of 1007 patients in the study population, 402 were diagnosed with stage II CRC and 605 with stage III CRC. Our study population was predominantly white (92.3%). Age at diagnosis ranged from 17 to 98 and the mean age at diagnosis for stage II and III patients was 65.4 ± 13.5 years and 60.4 ± 13.6 years, respectively (*p* < 0.001). There were more males with stage III CRC in comparison to stage II (61.3% vs. 56.2%). Telomere length was shorter in stage II patients compared to stage III (*p* = 0.0005). However, this difference was mainly driven by the difference in age distribution. The median follow-up time for our study population was 6.98 (IQR = 2.13, 11.4) years. A total of 32.2% (*n* = 324) patients died during the follow-up period. Overall survival after diagnosis was 0.882 (95% CI = 0.861–0.904) at 3 years and 0.791 (95% CI = 0.764–0.820) at 5 years and was higher for stage II patients (*p* = 0.09, log-rank test). Disease-free survival estimates were 0.819 (95% CI = 0.794–0.845) at 3 years and 0.726 (95% CI = 0.696–0.757) at 5 years and were higher for stage II patients (*p* = 0.002, log-rank test). Of the 1007 patients, 10.5% developed tumor recurrence (95% CI = 8.4–12.5%) at 3 years and 13.5% (95% CI = 11.1–15.8%) at 5 years. Incidence of tumor recurrence was also higher for stage III patients (*p* < 0.0001, log-rank test).

### 3.1. Relationship of Telomere Length with Age, Sex and Disease Stage

**Figure 1:**
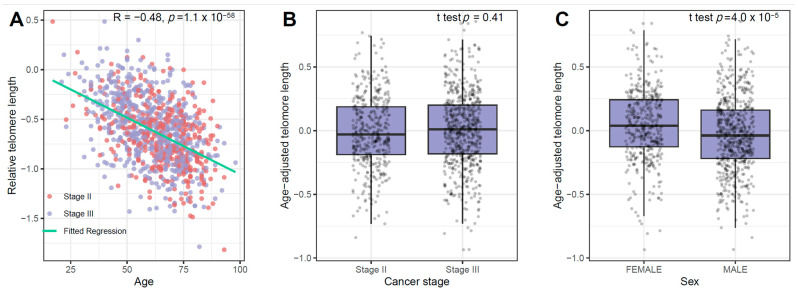
Relationship between relative LTL and age (**A**) and between age-adjusted relative LTL and cancer stage (**B**) and sex (**C**).

We first evaluated the influence of age on telomere length. A significant relationship was observed, with patients of older ages having shorter telomeres (Spearman correlation coefficient (r) = −0.48, 95%; *p* = 1.13 × 10^−58^) (Figure 1A). By taking the residuals from this model, age-adjusted telomere length was calculated and used to study additional influence of gender and disease stage (Figure 1B,C). These analyses show that females, in general, have significantly longer LTL than males (*p* = 3.97 × 10^−5^). However, the differences in LTL between stage II and stage III patients were not significant after age adjustment. We thus further removed the sex effects on telomere length based on linear regression. The age/sex-adjusted telomere length was next investigated for its association with genetic variation in telomere length-related genes.

### 3.2. Relationship of Age/Sex-Adjusted Telomere Length with SNPS in TERT, TERC, and OBFC1 Genes

**Figure 2:**
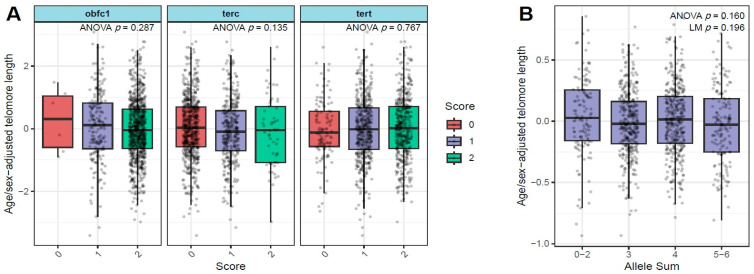
Relationship between relative LTL and SNPs in *TERT*, *TERC*, and *OBFC1* genes individually (**A**) and by allele sum (**B**).

Individually, the SNPs in *TERT*, *TERC,* and *OBFC1* did not have a strong influence on the age/sex adjusted telomere length (*p* = 0.77, 0.13, and 0.29, respectively, for the three SNPs) (Figure 2A). We combined the effect of these individual SNPs by calculating the allele sum ranging from 0 (no alleles) to 6 (all alleles present). There were no significant differences in age/sex-adjusted telomere length by allele sum, either as a continuous variable (*p* = 0.19) or as a categorical variable (*p* = 0.16) (Figure 2B).

### 3.3. Associations of Telomere Length with Overall Survival (OS) and Disease-Free Survival (DFS) in Combined Stage II and III Population of CRC Patients

**Figure 3:**
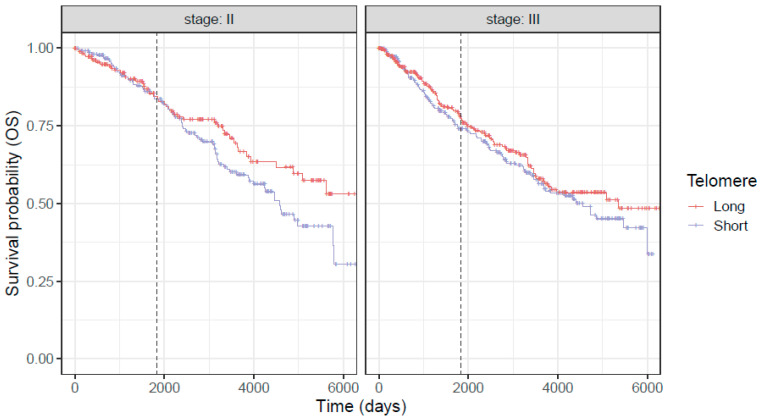
Kaplan–Meier survival curves for overall survival in stage II and III patients. Age/sex-adjusted telomere length dichotomized based on the median for visual comparison purposes. The vertical dashed line represents the five year survival time point following cancer diagnosis.

**Figure 4:**
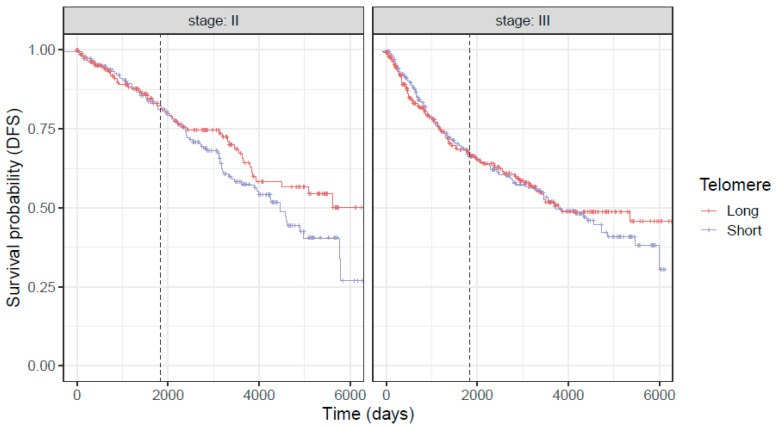
Kaplan–Meier curves for disease-free survival in stage II and III patients. Age/sex-adjusted telomere length is dichotomized based on the median for visual comparison purposes. The vertical dashed line represents the five year survival time point following cancer diagnosis.

The association between age/sex-adjusted telomere length and patient survival was evaluated using multi-variable Cox regression model. Covariates including age, sex, stage, and genotype were adjusted in the analysis. The analysis revealed that age/sex-adjusted telomere length is significantly and independently associated with both OS (*p* = 0.009) and DFS (*p* = 0.044) (Figure 3 and Figure 4).

Further, SNPs/alleles in *TERC* and *OBFC1* genes were associated with higher OS (*p* = 0.017 for *TERC* and 0.016 for *OBFC1*, respectively) (Table 2). Furthermore, the age and stage of the individual significantly correlated with OS and DFS. We also studied these relationships separately in stage II and III patients (Table 3 and Table 4). Age/sex-adjusted telomere length and SNPs in *TERC* and *OBFC1* genes are more significantly associated with OS and DFS in stage II patients than stage III patients.

To assess the robustness of the observed associations, we conducted a series of sensitivity analyses using multivariable Cox regression models by (1) categorizing age- and sex-adjusted telomere length into quartiles; (2) further adjusting for additional covariates, including BMI, smoking status, treatment, and comorbidities (hypertension and diabetes); and (3) excluding early death events (deaths occurring within six months). Across these analyses, the associations remained largely consistent (Supplementary Tables S1–S3).

## 4. Discussion

Within cancer cells themselves, the aggressiveness of cancer cells can be directly associated with whether a person is able to survive cancer and thus have a longer life following a cancer diagnosis One contributor to the aggressiveness of cancer cells may be related to the telomere length of the cancer cell DNA and the activation of telomere maintenance pathways that permits damaged tumor DNA to become immortalized and escape crisis and cell death. Telomere length is also a determinant and indicator of overall cellular fitness of not only the tumor, but also of the immune and metabolic health of the person with cancer. With such a rationale, we set out to investigate whether there is any relationship between a patient’s white blood cells’ telomere length, representing the patient’s overall health, and their ability to survive CRC.

Our results show that LTL was predictive of OS and DFS for both stage II and III CRC patients, particularly over a longer follow-up, such that patients with longer telomere lived longer than patients with short telomeres, and SNPs in *TERC* and *OBFC1* genes are associated with patient outcomes independent of telomere length. These findings suggest that LTL and these SNPs can serve as a predictor of outcome for patients undergoing treatment for CRC.

Multiple studies have been conducted to address the potential association between LTL and outcomes in CRC patients that yielded varied findings. For example, Chen et al. had reported an association of shorter LTL with worse OS and DFS in CRC patients, similar to our findings [38]. A recent study by Pauleck et al. reported similar trends, but statistical significance was not reached, probably because of the small sample size [39,40]. In contrast, Svenson et al. observed that shorter LTL predicted a higher OS, although it did not reach statistical significance as an independent prognostic indicator [41]. Our results show a direct association between longer LTL with higher OS and DFS in the overall population of patients with stage II and III CRC. One notable difference in our study from the previous investigations was the inclusion of patients with either stage II or III CRC, compared to other studies encompassing patients with stage I through IV CRC. The mechanism underlying the correlation between LTL and patient outcomes could be attributed to the immune function of patients with longer telomere length, as highlighted in previous studies [42,43,44]. Immunosenescence brought on by shortening telomeres could allow uncontrolled replication of cancer cells and decrease patients’ survival.

Even though short telomeres imply ‘aged’ ’senescent’ status, and therefore metabolically less activity than ‘younger’ cells, and even though cancer cells are known to usually have shorter telomeres than normal cells, cancer cells employ a strategy (telomerase activation, for most colorectal cancers) that helps maintain short telomeres and prevent them from shortening further [45,46,47]. Such a strategy equips the cancer cell to proliferate almost ad infinitum, accumulate new mutations and become more aggressive and drug-resistant, influencing survival. Since our results indicate the prognosticative value of LTL, telomere length measurements can be expected to have implications for management of CRC patients. For example, patients with short LTL may be more aggressively monitored compared to current approaches and may also be considered for more aggressive treatment regimens.

Further, our results also indicate that specific forms (because of SNPs) of some other genes also contribute to the length of telomeres in leukocytes. We focused on genetic variation in *TERT*, *TERC*, and *OBFC1* because these loci represent the core machinery governing telomere maintenance and together explain the largest and most reproducible fraction of heritable variation in leukocyte telomere length. *TERT* encodes the catalytic reverse transcriptase of telomerase, *TERC* provides the RNA template for telomere extension, and *OBFC1* (*STN1*) is a key component of the CST complex that regulates telomere replication and genome stability. Although recent GWASs have identified additional telomere-associated loci, these signals generally have smaller effect sizes and less direct mechanistic interpretability. By prioritizing these three canonical loci, we aimed to test a biologically grounded hypothesis that directly links fundamental telomere maintenance pathways to survival while minimizing heterogeneity introduced by weaker or indirect genetic signals. Beyond their role in regulating LTL, genetic variation at *TERC* and *OBFC1* may influence survival through telomere-independent biological pathways. *TERC* encodes the RNA template of telomerase and has emerging non-canonical functions in regulating DNA damage responses, mitochondrial homeostasis, and inflammatory signaling, including modulation of NF-κB and p53 pathways, which can directly affect tumor progression and host resilience. Experimental studies have shown that *TERC* can promote cellular proliferation and stress tolerance even in the absence of telomere elongation, suggesting that inherited variation at this locus may shape cancer outcomes through altered transcriptional and metabolic programs. *OBFC1* (*STN1*), a core component of the CST complex, plays a critical role in replication fork stability and genome integrity by coordinating laggingstrand synthesis and protecting stalled replication forks; dysfunction in this pathway can lead to replication stress, chromosomal instability, and impaired DNA repair capacity, all of which are key determinants of treatment response and survival. Thus, genetic variation at *TERC* and *OBFC1* may affect survival by altering cellular stress responses, immune function, and genomic stability, providing a biologically plausible explanation for the observed associations that are not fully mediated by measured LTL.

Our findings may appear counterintuitive at first, as the telomere-associated SNPs examined were not significantly associated with measured LTL in our cohort yet were associated with overall and disease-free survival. However, this pattern is consistent with prior evidence highlighting the complex and sometimes paradoxical role of telomere biology in colorectal cancer. For example, Jones et al. [32] reported that common genetic variation at *TERC* is associated with both longer telomeres and an increased risk of CRC. In this context, our results are biologically plausible and suggest that these germline variants may capture lifelong telomere maintenance capacity or broader telomere-related cellular processes—such as reduced cellular senescence or apoptosis—that are not fully reflected by a single post-diagnostic measurement of LTL in leukocytes. Furthermore, measured LTL in cancer patients may be influenced by disease status, treatment exposure, inflammation, and other post-diagnostic factors, potentially attenuating or obscuring underlying genetic effects. Taken together, our findings support an emerging view that telomere-associated genetic variation can influence CRC prognosis through mechanisms that are not fully mediated by measured LTL, consistent with pleiotropic effects of telomere biology genes. To assess the robustness of our findings, we performed additional analyses adjusting for available clinical comorbidities, including BMI, smoking status, diabetes, hypertension, and treatment (Supplementary Table S2). These extended models yielded association estimates that were highly consistent with those from the primary analyses, indicating that the observed relationships between telomere length and survival outcomes are not materially confounded by these factors. Although the association with disease-free survival was modestly attenuated after adjustment and did not reach the conventional significance threshold, the effect size remained similar, suggesting that this change is likely attributable to reduced statistical power from inclusion of multiple covariates with limited relevance. Overall, these results support the robustness of our conclusions with respect to adjustment for additional clinical variables.

### Study Limitations

Our study had some limitations. The different techniques for measuring telomeres may influence the telomere length results. Currently, there is no single standard for measurement of telomere length. We used qPCR for measuring telomere length, which utilizes average cumulative amount of TTAGGG repeats relative to the diploid human genome. Further, qPCR of telomere length measures both the telomeric duplex and the G-rich overhang, which is important to telomere capping. It has also been suggested that qPCR can minimize the variability between samples that can occur when using different restriction enzymes to assess telomere length by telomere restriction fragment (TRF) analysis. Southern blotting measures telomere length with higher resolution and precision than qPCR, but its requirement for much higher input genomic DNA, being both time- and labor- intensive, and the risk for Southern blotting to overestimate telomere length make it more challenging to apply to larger scale studies than qPCR. However, the fact that our result shows the expected significant association between decrease in telomere length with age should imply confidence in our methodological approach and, correspondingly, on our overall conclusions. In this regard, we have begun developing and implementing sequence-based evaluation of telomere length (and composition) to most accurately relate LTL with prognostic parameters of CRC patients.

Also, while our study included patients with stage II and III CRC, it is imperative to improve treatment recommendations for these patients and to perform large-cohort studies that include CRC patient populations from all stages that may yield new TL dynamics not observed in our study.

Our study population predominantly included a white population who reside in the United States, and it has been shown that the race of an individual influences telomere length [48]. For example, black people at birth have been found to have longer telomeres when compared with a white population [49], even though the rate of attrition through the lifespan is higher in this population [50]. Thus, studies encompassing patients from diverse racial and possibly ethnic groups may shed new light on this relationship and provide additional insights on the role of telomere length in patients with CRC.

Lastly, our study did not adjust for other potential confounders in patient outcomes like cardiovascular disease, hypertension, and diabetes mellitus. These clinical variables could also be responsible for the significant association of age with patient outcomes, independently or in conjunction with telomere length.

## 5. Conclusions

We show that the prognosis of patients diagnosed with stage II and III CRC is significantly influenced by their telomere length in peripheral blood leukocytes. We also show that other genes (via SNPs) also influence LTL, implying that maintenance of telomeres is a complex multi-gene process. The association of *TERC* and *OBFC1* genes with patient outcomes independent of telomere length is intriguing, requires further studies, and implies that these genes have telomere-independent roles on physiological processes that influence aggressiveness of CRC cells. Development of a prognostication model incorporating LTL and these *TERC* and *OBFC1* SNPs could improve counseling, surveillance and management approaches for patients with CRC.

## Figures and Tables

**Table 1:** Baseline characteristics of the study population by cancer stages.

Patient Characteristics	Stage II (*N* = 402)	Stage III (*N* = 605)	Total (*N* = 1007)	*p*-Value
Sex, *n* (%)				0.106
Female	176 (43.8%)	234 (38.7%)	410 (40.7%)	
Male	226 (56.2%)	371 (61.3%)	597 (59.3%)	
Age at Dx				<0.0001
Mean (SD)	65.4 (13.51)	60.4 (13.61)	62.4 (13.78)	
Median	67.0	61.0	64.0	
Range	17.0, 93.0	21.0, 98.0	17.0, 98.0	
Race, *n* (%)				0.307
Asian	1 (0.2%)	5 (0.8%)	6 (0.6%)	
Black	4 (1.0%)	0 (0.0%)	4 (0.4%)	
Other	6 (1.5%)	9 (1.5%)	15 (1.5%)	
Unknown	18 (4.5%)	33 (5.5%)	51 (5.1%)	
White	373 (92.8%)	558 (92.2%)	931 (92.5%)	
Status death, *n* (%)				0.462
No	278 (69.2%)	405 (66.9%)	683 (67.8%)	
Yes	124 (30.8%)	200 (33.1%)	324 (32.2%)	
Allele group, *n* (%)				0.310
0–2	50 (12.4%)	81 (13.4%)	131 (13.0%)	
3	131 (32.6%)	215 (35.5%)	346 (34.4%)	
4	163 (40.5%)	210 (34.7%)	373 (37.0%)	
5–6	58 (14.4%)	99 (16.4%)	157 (15.6%)	

**Table 2:** Cox regression model for overall survival and disease-free survival in combined stage II and III patients in relation to SNPs in the *TERT*, *TERC,* and *OBFC1* genes. All variables were significant, except *TERT*.

Overall Survival Model			Disease-Free Survival Model	
Patient Characteristics	Log Hazard Ratio	*p*-Value	Log Hazard Ratio	*p*-Value
Genotype				
*TERT*	−0.00827	0.920	0.0198	0.797
*TERC*	−0.237	0.017	−0.208	0.023
*OBFC1*	−0.314	0.016	−0.207	0.093
Male sex	0.284	0.015	0.192	0.077
Age	0.0531	<2 × 10^−16^	0.0383	<2 × 10^−16^
Stage III	0.462	8.34 × 10^−5^	0.531	1.77 × 10^−6^
Sex/Age-adjusted telomere length	−0.550	0.009	−0.386	0.044

**Table 3:** Cox regression model for overall survival and disease-free survival in stage II patients in relation to SNPs in the *TERT*, *TERC,* and *OBFC1* genes.

Overall Survival Model			Disease-Free Survival Model	
Patient Characteristics	Log Hazard Ratio	*p*-Value	Log Hazard Ratio	*p*-Value
Genotype				
*TERT*	−0.0333	0.805	0.0201	0.878
*TERC*	−0.330	0.059	−0.359	0.033
*OBFC1*	−0.547	0.015	−0.403	0.069
Male sex	0.407	0.029	0.389	0.031
Age	0.0610	1.03 × 10^−10^	0.0496	1.33 × 10^−8^
Sex/Age-adjusted telomere length	−0.794	0.017	−0.758	0.018

**Table 4:** Cox regression model for overall survival and disease-free survival in stage III patients in relation to SNPs in the *TERT*, *TERC*, and *OBFC1* genes. Age was the only significant variable for both overall survival and disease-free survival.

Overall Survival Model			Disease-Free Survival Model	
Patient Characteristics	Log Hazard Ratio	*p*-Value	Log Hazard Ratio	*p*-Value
Genotype				
*TERT*	0.0119	0.909	0.0311	0.748
*TERC*	−0.179	0.138	−0.130	0.236
*OBFC1*	−0.223	0.172	−0.161	0.281
Male sex	0.208	0.165	0.0926	0.497
Age	0.0488	5.22 × 10^−15^	0.0325	1.2 × 10^−9^
Sex/Age-adjusted telomere length	−0.429	0.111	−0.225	0.349

## Data Availability

The original contributions presented in this study are included in the article/Supplementary Material. Further inquiries can be directed to the corresponding authors.

## Supplementary Materials

The following supporting information can be downloaded at: https://www.mdpi.com/article/10.3390/cancers18030490/s1, Table S1: Cox regression model categorizing the association of overall survival (OS) and disease free survival (DFS) with age and sex-adjusted telomere length categorized into quartiles; Table S2: Cox Regression Model for the association of age and sex adjusted telomere length (tlen.adj.sex) and overall survival (OS) and disease-free survival (DFS) adjusted for additional comorbidities including body mass index, diabetes, hypertension, smoking exposure, cancer treatment type; Table S3: Cox Regression Model for the association of age and sex adjusted telomere length(tlen.adj.sex) and overall survival (OS) and disease-free survival (DFS) excluding early deaths within the first six months following treatment.
