# Supplementary material for: Leukocyte Telomere Length Variants Are Independently Associated with Survival of Patients with Colorectal Cancer
Source: Cancers (Basel). 2026 Feb 2;18(3):490. doi: 10.3390/cancers18030490 (PMC12897216; doi:10.3390/cancers18030490)
Supplement: Supplementary file 1 [file cancers-18-00490-s001.zip › cancers-4026381-supplementary.pdf]

Supplementary Tables

Table S1. Cox regression model categorizing the association of overall survival (OS) and disease free survival (DFS) with age and sex- adjusted telomere length categorized into quartiles. Tlen_ qQ2 = telomere length in quartile 2 of the overall telomere lengths; qQ3=quartile 3; qQ4= quartile 4.

Variable	OS: HR (95% CI)	OS: p-value (FDR)	DFS: HR (95% CI)	DFS: p-value (FDR)
**age**	1.054 (1.043-1.065)	<0.001 (<0.001)	1.039 (1.03-1.048)	<0.001 (<0.001)
**obfc1**	0.758 (0.586-0.981)	0.035 (0.053)	0.825 (0.648-1.051)	0.119 (0.215)
**sex: MALE**	1.323 (1.053-1.662)	0.016 (0.045)	1.206 (0.976-1.491)	0.083 (0.186)
**stage: Stage III**	1.607 (1.274-2.025)	<0.001 (<0.001)	1.708 (1.373-2.126)	<0.001 (<0.001)
**terc**	0.795 (0.656-0.965)	0.02 (0.045)	0.818 (0.684-0.978)	0.027 (0.082)
**tert**	0.998 (0.849-1.172)	0.977 (0.977)	1.026 (0.881-1.195)	0.741 (0.797)
**tlen_qQ2**	0.889 (0.663-1.192)	0.432 (0.491)	0.964 (0.73-1.274)	0.797 (0.797)
**tlen_qQ3**	0.703 (0.515-0.961)	0.027 (0.049)	0.861 (0.647-1.146)	0.304 (0.457)
**tlen_qQ4**	0.886 (0.654-1.201)	0.437 (0.491)	0.916 (0.686-1.223)	0.553 (0.711)

Table S2. Cox Regression Model for the association of age and sex adjusted telomere length (tlen.adj.sex) and overall survival (OS) and disease-free survival (DFS) adjusted for additional comorbidities including body mass index, diabetes, hypertension, smoking exposure, cancer treatment type. HR= Hazard ratio, FDR = false discovery rate.

Variable	OS: HR (95% CI)	OS: p-value (FDR)	DFS: HR (95% CI)	DFS: p-value (FDR)
age	1.053 (1.042-1.065)	<0.001 (<0.001)	1.037 (1.027-1.047)	<0.001 (<0.001)
Body Mass Index (BMI)	1.003 (0.983-1.024)	0.761 (0.815)	0.998 (0.979-1.017)	0.819 (0.877)
diabetes: yes	0.854 (0.655-1.114)	0.244 (0.305)	0.854 (0.664-1.097)	0.216 (0.27)
hypertension: Yes	0.985 (0.766-1.266)	0.904 (0.904)	1.037 (0.818-1.314)	0.765 (0.877)
obfc1	0.725 (0.559-0.939)	0.015 (0.025)	0.781 (0.612-0.996)	0.047 (0.1)
sex: Male	1.341 (1.064-1.691)	0.013 (0.024)	1.223 (0.986-1.517)	0.067 (0.103)
smoking: yes	1.353 (0.876-2.089)	0.173 (0.236)	1.337 (0.9-1.987)	0.151 (0.205)
stage: Stage III	1.832 (1.409-2.382)	<0.001 (<0.001)	1.964 (1.533-2.517)	<0.001 (<0.001)
terc	0.81 (0.666-0.986)	0.036 (0.053)	0.837 (0.699-1.003)	0.053 (0.1)
tert	0.963 (0.818-1.134)	0.651 (0.751)	0.989 (0.848-1.153)	0.888 (0.888)
tlen.adj.sex	0.857 (0.761-0.966)	0.011 (0.024)	0.903 (0.809-1.008)	0.069 (0.103)
Surgery only	0.253 (0.156-0.412)	<0.001 (<0.001)	0.227 (0.146-0.353)	<0.001 (<0.001)
Surgery & chemotherapy	0.202 (0.119-0.342)	<0.001 (<0.001)	0.198 (0.122-0.32)	<0.001 (<0.001)
Surgery & radiochemotherapy	0.205 (0.122-0.346)	<0.001 (<0.001)	0.182 (0.113-0.292)	<0.001 (<0.001)
treatment: not specified	0.286 (0.13-0.628)	0.002 (0.005)	0.238 (0.114-0.496)	<0.001 (<0.001)

Table S3. Cox Regression Model for the association of age and sex adjusted telomere length(tlen.adj.sex) and overall survival (OS) and disease-free survival (DFS) excluding early deaths within the first six months following treatment. HR=hazard ratio, FDR=false discovery rate.

Variable	OS: HR (95% CI)	OS: p-value (FDR)	DFS: HR (95% CI)	DFS: p-value (FDR)
**age**	1.053 (1.042-1.064)	<0.001 (<0.001)	1.037 (1.028-1.047)	<0.001 (<0.001)
**obfc1**	0.72 (0.554-0.936)	0.014 (0.024)	0.809 (0.632-1.034)	0.09 (0.114)
**sex: MALE**	1.317 (1.044-1.663)	0.02 (0.028)	1.2 (0.967-1.489)	0.098 (0.114)
**stage: Stage III**	1.577 (1.246-1.995)	<0.001 (<0.001)	1.697 (1.359-2.119)	<0.001 (<0.001)
**terc**	0.806 (0.663-0.981)	0.032 (0.037)	0.828 (0.691-0.992)	0.041 (0.071)
**tert**	0.97 (0.824-1.142)	0.716 (0.716)	1.002 (0.859-1.168)	0.985 (0.985)
**tlen.adj.sex**	0.844 (0.748-0.953)	0.006 (0.014)	0.888 (0.795-0.992)	0.036 (0.071)
